# SMAD4 loss is associated with response to neoadjuvant chemotherapy plus hydroxychloroquine in patients with pancreatic adenocarcinoma

**DOI:** 10.1111/cts.13029

**Published:** 2021-05-18

**Authors:** Naomi Fei, Sijin Wen, Rajesh Ramanathan, Melissa E. Hogg, Amer H. Zureikat, Michael T. Lotze, Nathan Bahary, Aatur D. Singhi, Herbert J. Zeh, Brian A. Boone

**Affiliations:** ^1^ Division of Hematology/Oncology Department of Medicine West Virginia University Morgantown West Virginia USA; ^2^ Department of Biostatistics School of Public Health West Virginia University Morgantown West Virginia USA; ^3^ Department of Surgery Banner MD Anderson Cancer Center Phoenix Arizona USA; ^4^ Division of Surgical Oncology Department of Surgery Northshore University Health System Chicago Illinois USA; ^5^ Division of Surgical Oncology Department of Surgery University of Pittsburgh Pittsburgh Pennsylvania USA; ^6^ Division of Hematology/Oncology Department of Medicine University of Pittsburgh Pittsburgh Pennsylvania USA; ^7^ Department of Pathology University of Pittsburgh Pittsburgh Pennsylvania USA; ^8^ Division of Surgical Oncology Department of Surgery UT Southwestern Dallas Texas USA; ^9^ Division of Surgical Oncology Department of Surgery West Virginia University Morgantown West Virginia USA; ^10^ Department of Microbiology, Immunology and Cell Biology West Virginia University Morgantown West Virginia USA

## Abstract

*SMAD4*, a tumor suppressor gene, is lost in up to 60%–90% of pancreatic adenocarcinomas (PDAs). Loss of *SMAD4* allows tumor progression by upregulating autophagy, a cell survival mechanism that counteracts apoptosis and allows intracellular recycling of macromolecules. Hydroxychloroquine (HCQ) is an autophagy inhibitor. We studied whether HCQ treatment in *SMAD4* deficient PDA may prevent therapeutic resistance induced by autophagy upregulation. We retrospectively analyzed the SMAD4 status of patients with PDA enrolled in two prospective clinical trials evaluating pre‐operative HCQ. The first dose escalation trial demonstrated the safety of preoperative gemcitabine with HCQ (NCT01128296). More recently, a randomized trial of gemcitabine/nab‐paclitaxel +/− HCQ evaluated Evans Grade histopathologic response (NCT01978184). The effect of SMAD4 loss on response to HCQ and chemotherapy was studied for association with clinical outcome. Fisher’s exact test and log‐rank test were used to assess response and survival. Fifty‐two patients receiving HCQ with neoadjuvant chemotherapy were studied. Twenty‐five patients had SMAD4 loss (48%). 76% of HCQ‐treated patients with SMAD4 loss obtained a histopathologic response greater than or equal to 2A, compared with only 37% with SMAD4 intact (*p *= 0.006). Although loss of SMAD4 has been associated with worse outcomes, in the current study, loss of SMAD4 was not associated with a detriment in median overall survival in HCQ‐treated patients (34.43 months in SMAD4 loss vs. 27.27 months in SMAD4 intact, *p *= 0.18). The addition of HCQ to neoadjuvant chemotherapy in patients with PDA may improve treatment response in those with SMAD4 loss. Further study of the relationship among SMAD4, autophagy, and treatment outcomes in PDA is warranted.

**Study Highlights**

**WHAT IS THE CURRENT KNOWLEDGE ON THE TOPIC?**

​

*SMAD4* is depleted in 60%–90% of pancreatic adenocarcinomas (PDAs) and associated with poor prognosis. SMAD4‐deficient PDA cells are resistant to therapies by upregulating autophagy, a cell survival mechanism that allows recycling of organelles during cytotoxic stress.

**WHAT QUESTION DID THIS STUDY ADDRESS?**

​

This study examined clinical outcomes after autophagy inhibition with hydroxychloroquine (HCQ) in patients with PDA according to SMAD4 status. We hypothesized that patients with depleted SMAD4 would derive the greatest benefit from HCQ.

**WHAT DOES THIS STUDY ADD TO OUR KNOWLEDGE?**

​

Patients with SMAD4 depleted PDA had a significant improvement in histopathologic response and R0 resection rates after receiving HCQ compared with patients with preserved SMAD4. When treated with HCQ, loss of SMAD4 was not associated with a detriment in median overall survival.

**HOW MIGHT THIS CHANGE CLINICAL PHARMACOLOGY OR TRANSLATIONAL SCIENCE?**

​

In patients with SMAD4 loss, the addition of HCQ to neoadjuvant chemotherapy is associated with improved clinical outcomes. Further study of autophagy inhibition with HCQ in PDA with SMAD4 loss is warranted.

​

## INTRODUCTION

Pancreatic cancer has the third‐highest cancer related mortality in the United States and is destined to be the second by 2025.^1^ Despite recent advances in available therapies, median overall survival (OS) of patients with pancreatic cancer is less than 6 months, and 5‐year survival is less than 10%.^2^, ^3^ This dismal prognosis is driven by early metastatic spread and resistance to treatment, promoted by a unique tumor microenvironment. Pancreatic cancers rely on autophagy as a survival mechanism whereby damaged organelles are recycled and used for energy during metabolic stress.^4^ Pancreatic cancer cells utilize autophagy to support the abnormal nutrient demands of rapid growth in a hypoxic, acidotic tumor microenvironment.^5^, ^6^, ^7^ Autophagy also allows malignant cells to escape the cellular damage incurred by chemotherapy and radiation treatments.^8^, ^9^, ^10^ Beyond metabolic recycling as a tumor survival mechanism, autophagy may also promote tumor growth through other mechanisms. Autophagy also promotes formation of dense stroma by cancer‐associated fibroblasts, hindering the cytotoxic effects of chemotherapy on cancer cells.^11^ Higher levels of autophagy correlate with worse prognosis in pancreatic cancer.^12^

Inhibition of autophagy promotes apoptosis and represents a novel treatment target in pancreatic cancer.^13^, ^14^, ^15^ Hydroxychloroquine (HCQ) is an inexpensive, orally available, well‐tolerated medication that inhibits the final step of autophagy and therefore may potentiate antineoplastic therapies.^16^, ^17^ A recent phase I/II clinical trial added high‐dose HCQ to neoadjuvant gemcitabine in patients with localized pancreatic adenocarcinoma. The combination was safe and well‐tolerated with no dose‐limiting toxicity. Seventy‐seven percent of patients achieved R0 resection, which was superior when compared with historical controls. Patients who had a cancer antigen (CA) 19‐9 response to treatment also had improved OS and disease‐free survival (DFS).^18^ A follow‐up, randomized phase II clinical trial of HCQ added to pre‐operative gemcitabine and nab‐paclitaxel in patients with potentially resectable tumors noted that Evans grade histopathologic and CA 19‐9 biomarker responses were significantly improved in patients receiving HCQ.^19^ The success of these early phase trials suggests a potential benefit to HCQ autophagy inhibition in pancreatic cancer.

*SMAD4*, a tumor suppressor gene, is mutated or deleted in approximately 55% of pancreatic cancers.^20^ Loss of SMAD4 is associated with pancreatic tumor progression, metastases,^12^, ^21^ and is an important negative prognostic factor for OS.^22^, ^23^ Increased levels of autophagy have been observed in pancreatic cancer cells with loss of SMAD4 and SMAD4‐mediated autophagy has been implicated in treatment resistance in pancreatic cancer.^3^

Because SMAD4 mutated or deleted pancreatic cancers have an increased reliance on autophagy for treatment resistance, we hypothesized that patients with SMAD4 tumor loss/mutation would derive the greatest benefit from autophagy inhibition with HCQ. In this retrospective analysis of two sequential prospective clinical trials, patients who previously received HCQ with neoadjuvant chemotherapy were evaluated according to SMAD4 status for associations with survival, Evans grade histopathologic response, R0 resection rates, and CA 19‐9 biomarker response.

## METHODS

### Study design

This was a retrospective analysis of two prospective clinical trials evaluating HCQ in the pre‐operative setting for patients with pancreatic cancer.^4^, ^24^ Institutional review board approval was obtained from the University of Pittsburgh for the clinical trials analyzed in the current work (PRO10010028 and PRO13080444). The trials were registered with the National Cancer Institute (NCT01128296 and NCT01978184). Patients included in these prospective trials had not previously been treated with HCQ or received chemotherapy. Upon trial enrollment, medication review was conducted to ensure appropriate tolerance of HCQ and chemotherapy. All patients signed informed consent prior to participation. Both trial protocols and consent forms included approval for analysis of tissue specimens and correlation with oncologic outcomes as performed in the current study. The first trial was a safety phase dose escalation (UPCI 09‐122, NCT01128296) demonstrating safety and tolerability of 1 month of pre‐operative gemcitabine with up to 1200 mg/day of HCQ. Patients who were treated with less than the maximum tolerated dose of HCQ (600 mg b.i.d.) during the dose escalation phase were excluded from the current analysis. This was followed with a randomized trial of 2 months of gemcitabine/nab‐paclitaxel with or without 600 mg twice daily of HCQ in the pre‐operative setting (UPCI 13‐074, NCT01978184) that demonstrated a significant increase in histopathologic and biochemical responses in patients receiving HCQ.

### Immunohistochemical analysis of SMAD4 expression

**FIGURE 1:**
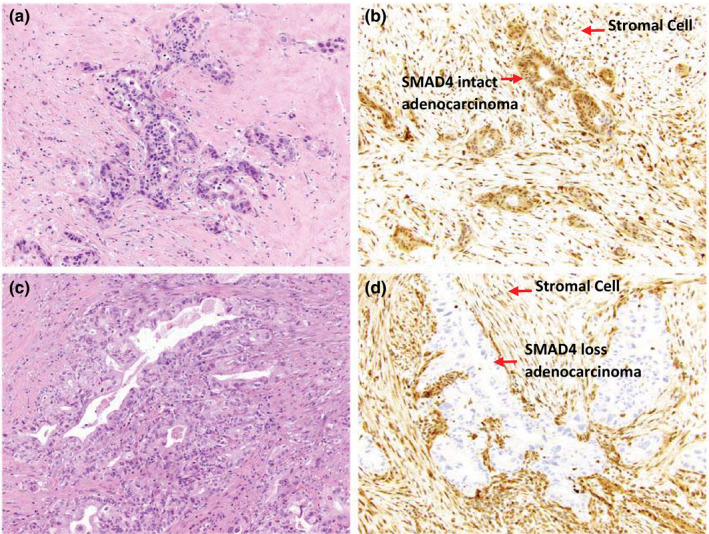
Representative images of SMAD4 staining. Representative hematoxylin‐eosin staining in (a) and (c) of pancreatic cancer specimens. SMAD4 was scored intact with strong nuclear and cytoplasmic staining in >10% of malignant cells compared to stromal control cells (b) or lost with lack of staining in both the nuclear and cytoplasmic compartments compared to stromal control cells (d)

Assessment of SMAD4 was performed blinded to any other patient data, including outcome. Standard automated immunohistochemical labeling on formalin‐fixed, paraffin‐embedded, 4 μm thick tissue sections was performed for SMAD4 (clone B‐8, 1:500; Santa Cruz Biotechnology, Dallas, TX). Following deparaffinization with serial xylene treatments and rehydration in ethanol, the slides were stained using the Ventana BenchMark XT; the enzymatic reactivity was visualized with the iVIEW DAB Detection Kit (Ventana Medical Systems, Tucson, AZ). The volume of fixative is at least 15–20 times that of the volume of tissue. College of American Pathologists guidelines of a minimum of 6 h and maximum of 96 h were followed, but the fixative duration varies from specimen to specimen. Immunohistochemical scoring of SMAD4 expression was performed similar to those published previously.^21^, ^25^ Normal SMAD4 staining of stromal cells surrounding the malignant glands were used as an internal positive control. The SMAD4 staining was scored as follows: intact (strong nuclear and cytoplasmic staining in >10% of cells; Figure 1b) or lost (lack of staining in both the nuclear and cytoplasmic compartments; Figure 1d). Representative hematoxylin‐eosin staining for pancreatic tumors are show in Figure 1a and c.

### Statistical analysis

Data analysis was performed using SAS 9.1 (SAS Institute, Cary, NC) and R (version 3.6.3; R Foundation, Vienna, Austria). Descriptive statistical analyses were performed to summarize patient’s characteristics, including summary tables, proportions, median, means, and SDs. Fisher exact test was used in the data analysis of SMAD4 status with other categorical variables, whereas the Wilcoxon rank sum test was used in the data analysis of SMAD4 status with continuous variables. Multivariable logistic regression modeling was performed adjusting for patient demographics. A leave‐one‐out cross‐validation analysis was performed for internal validation of the model to demonstrate accuracy given the small sample size. Kaplan‐Meier method and log‐rank test were used to examine OS and DFS by SMAD4 status. All statistical tests were 2‐sided and *p* < 0.05 was considered statistically significant.

## RESULTS

### Patient selection

**FIGURE 2:**
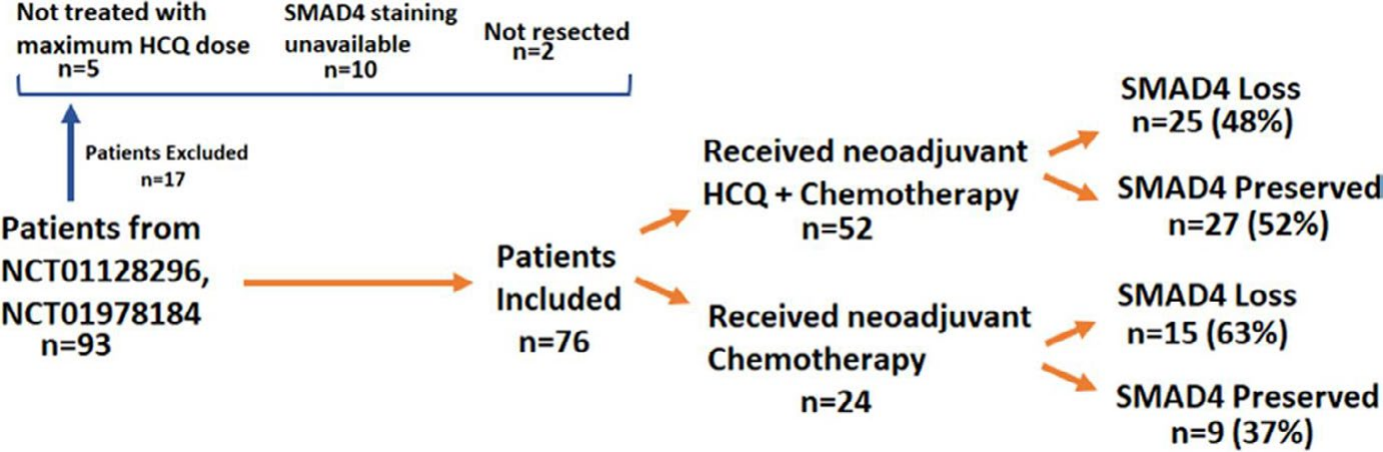
Patients enrolled in two prospective clinical trials, retrospectively stratified by SMAD4 status. HCQ, hydroxychloroquine

Of 93 patients enrolled in the prospective clinical trials, 17 patients were excluded from this analysis (Figure 2). Five patients were excluded as they were not treated with the maximum dose of HCQ during the dose escalation phase, 10 patients did not have SMAD4 staining performed, and 2 patients were not resected and therefore had no tumor available for SMAD4 staining. Of patients treated with HCQ as part of these trials, 25 of the 52 had SMAD4 loss (48%), compared with 15 of the 24 patients treated with chemotherapy alone (63%, *p *= 0.32). Patient demographics and clinical data are reported in Table 1. Male patients made up a significantly lower percentage of the cohort with SMAD4 loss (36% vs. 70%, *p *= 0.01). Body mass index was significantly higher in the cohort with SMAD4 loss (29.1 ± 6 vs. 26.3 ± 4, *p *= 0.05). No other demographic differences between SMAD4 groups were identified.

**TABLE 1:** Demographics stratified by SMAD4 status for patients treated with chemotherapy and HCQ

	SMAD4 preserved (*n* = 27)	SMAD4 loss (*n* = 25)	*p* value
Male *n* (%)	19 (70)	9 (36)	**0.01**
Age (SD)	66 ± 10	64 ± 8	0.19
Body mass index (SD)	26.3 ± 4	29.1 ± 6	**0.05**
Days from diagnosis to surgery (SD)	72 ± 19	82 ± 21	0.12
Pretreatment CA 19‐9 (SD)	1821.6 ± 2927	1697.3 ± 3660	0.45
CT vascular involvement (%)	10 (37)	11 (44)	0.61
EUS size in cm (SD)	2.77 ± 0.71	2.85 ± 0.86	0.64
EUS stage >2B (%)	15 (56)	17 (68)	0.64
Tumor size in cm (SD)	3.08 ± 1.37	2.65 ± 1.36	0.13
Adjuvant chemotherapy (%)	22 (81)	23 (92)	0.27
Tumor stage (%)			
1	1 (3.7)	2 (8)	0.69
2	4 (14.8)	3 (12)	
3	17 (63)	20 (80)	
4	1 (3.1)	n/a	
Nodal involvement (%)	17 (63)	17 (68)	0.7
Angiolymphatic invasion (%)	19 (70)	20 (80)	0.42
Perineural invasion (%)	24 (89)	20 (80)	0.49

Abbreviations: CA, cancer antigen; CT, computed tomography; EUS, endoscopic ultrasound; HCQ, hydroxychloroquine.

1

### Impact of SMAD4 status on outcomes for patients treated with HCQ

**FIGURE 3:**
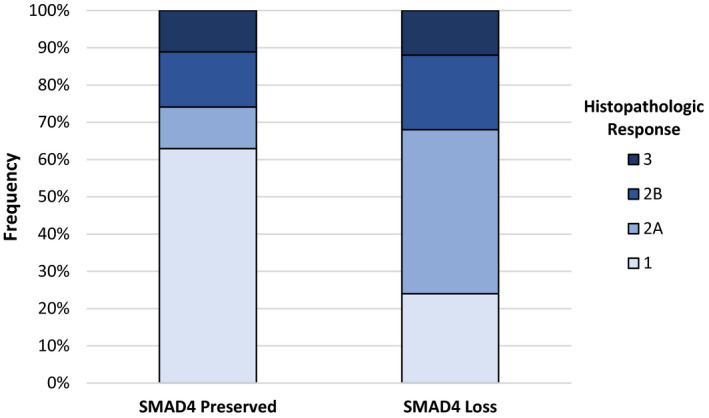
Evans Grade histopathologic response in hydroxychloroquine (HCQ)‐treated patients with pancreatic cancer stratified by SMAD4 status. Patients with loss of SMAD4 had significant higher histopathologic response to treatment than patients with SMAD4 intact

Among the patients treated with HCQ, a higher rate of Evans grade 2A or greater histopathologic response was noted in those with SMAD4 loss as compared with SMAD4 intact (76% vs. 37%, *p *= 0.006; Figure 3). Ninety‐two percent of patients with SMAD4 loss obtained an R0 resection compared with only 67% with intact SMAD4 (*p *= 0.04; Table 2). There were no significant differences in CA 19‐9 response between patients based on SMAD4 status. The improved histopathologic response in patients with SMAD4 loss persisted on multivariable regression analysis, demonstrating SMAD4 status as an independent predictor of histopathologic response (Table 2; *p *= 0.005). Cross‐validation analysis of the model demonstrated a concordance of 0.692 and kappa statistic of 0.39 (*p *= 0.007), validating the model accuracy given the small sample size.

**TABLE 2:** Outcomes stratified by SMAD4 status in HCQ‐treated patients

	SMAD4 preserved (*n* = 27)	SMAD4 loss (*n* = 25)	*p* value (univariate)	*p* value* (multivariate)
Evans Grade histopathologic response (%)				
1	17 (63)	6 (24)	**0.006**	**0.005** **
≥2A	10 (37)	19 (76)		
R0 resection (%)				
No	9 (33)	2 (8)	**0.039**	0.071
Yes	18 (67)	23 (92)		
Decrease in CA 19‐9 (%)				
<50%	7 (26)	4 (16)	0.40	0.47
≥50%–74%	3 (11)	6 (24)		
≥75%–89%	11 (40)	6 (24)		
≥90%	3 (11)	6 (24)		
N/A	3 (11)	3 (12)		
Percent decrease of CA 19‐9 (mean)	12.4	7.4	0.62	0.87
Median CA 19‐9 post‐treatment (IQR)	200.4 (39–547)	42.7 (28–385)	0.23	0.49
Median OS (months)	27.27	34.43	0.18	0.17
Median DFS (months)	13.23	15.43	0.49	0.41

Abbreviations: DFS, disease‐free survival; HCQ, hydroxychloroquine; IQR, interquartile range; N/A, not applicable; OS, overall survival.

* The *p* values were from multivariate models, adjusting for baseline data age, sex, and body mass index.

** Based on a leave‐one‐out cross validation analysis, the concordance statistic is 0.692, kappa statistic is 0.39 (*p* = 0.007); *p*<0.05 are bolded.

Patient outcomes stratified by SMAD4 status for the chemotherapy alone patients are shown in Table S1. Data from patients in both the chemotherapy with HCQ treatment group and the chemotherapy alone treatment group with SMAD4 loss and SMAD4 intact in shown in Tables S2 and S3, respectively. When comparing treatment groups, HCQ appeared to have a more significant effect in patients with SMAD4 loss, shown by a higher R0 resection rate and the suggestion of a detrimental effect in patients with SMAD4 preserved, reflected by a lower rate of greater than or equal to 2A histopathologic response that neared statistical significance.

### Assessment of survival in HCQ‐treated patients

DFS and OS curves for HCQ‐treated patients are reported in Figures S1 and S2, respectively. There was a nonsignificant trend toward improved median OS in patients treated with HCQ with SMAD4 loss (34.43 months vs. 27.27 months, *p *= 0.18). There were no significant differences in DFS. Survival curves for patients treated with chemotherapy alone from the control group, stratified by SMAD4 status, are shown in Figures S3 and S4. Consistent with existing literature, SMAD4 loss in patients treated with chemotherapy alone was associated with a trend toward worse survival outcomes.^12^, ^21^ Comparing these survival data from both treatment groups suggests that SMAD4 status had less of an impact on survival outcomes in HCQ‐treated patients.

### Assessing autophagy by SMAD4 status

Upregulation of autophagy has been identified in SMAD4 mutated or deleted pancreatic cancer cells.^3^ We investigated markers of autophagy regulation, including Beclin1 and ATG7, in resected tissue specimens. There were no significant differences in autophagy markers according to SMAD4 status.

## DISCUSSION

Autophagy is emerging as an increasingly important therapeutic target in pancreatic cancer. The tumor suppressor gene *SMAD4*, mutated or deleted in 55% of pancreatic cancer, has been implicated in treatment resistance via upregulation of autophagy.^3^, ^20^

During radiotherapy, high volumes of intracellular free radicals are generated, producing cytotoxic oxidative damage in cancer cells.^26^ Cancer cells demonstrate increased expression of autophagy‐related genes and accumulation of autophagosomes after radiation exposure.^27^ The recycling of organelles during autophagy serves as a rescue from radiation damage, perhaps contributing to radio‐resistance.^28^ Blockade of autophagy‐related genes results in radio‐sensitization of carcinoma cells.^27^ Pancreatic cancer cells with SMAD4 knockdown demonstrate increased levels of autophagy and enhanced tolerance to irradiation. Both the restoration of SMAD4 expression and inhibition of autophagy using chloroquine results in increased radiation sensitivity.^3^

Similar trends have been demonstrated during chemotherapy treatment. After treatment with gemcitabine, cellular markers of autophagy are upregulated.^9^ Studies in vitro and in vivo have proven that autophagy prevents pancreatic carcinoma cells from entering the apoptotic pathway after stimulus with gemcitabine, contributing to treatment resistance.^14^ Chloroquine and HCQ serve as late inhibitors of autophagy by preventing fusion of the autophagosome and lysosome to block recycling of organelles.^29^ As an inhibitor of autophagy, HCQ may improve tumor response to chemotherapy.^16^, ^17^ In glioblastoma and chronic myeloid leukemia, the addition of chloroquine has improved response to chemotherapeutics and tyrosine kinase inhibitors, respectively.^30^, ^31^ Given the autophagy‐mediated treatment‐resistance in SMAD4 mutated or deleted pancreatic cancer cells, this study retrospectively examined the effect of HCQ with neoadjuvant chemotherapy according to SMAD4 status.

The addition of neoadjuvant HCQ was associated with improved R0 resection rates and higher degree of histopathologic response in patients with SMAD4 loss compared with SMAD4 intact. Previous studies have noted improved pathologic response rate^19^ and overall response rate^32^ in patients receiving concurrent neoadjuvant HCQ and chemotherapy. This analysis is the first to suggest specific benefit in patients with SMAD4 loss. This may indicate a role for delivery of HCQ especially to patients with SMAD4 loss in order to improve tumor resectability and inform patient selection for future studies on HCQ or other emergent and experimental autophagy inhibitors.^32^, ^33^, ^34^, ^35^, ^36^

Both R0 resection and histopathologic response have been associated with improved survival in pancreatic cancer.^37^ Although the clinical studies examined were not sufficiently powered to identify survival benefit, the observed trends are of interest. Loss of SMAD4 is generally associated with decreased OS,^22^, ^23^ whereas in patients treated with HCQ as part of these studies, survival trends were similar regardless of SMAD4 status. SMAD4 loss also did not appear to be associated with a detriment in DFS in patients receiving HCQ. A study in patients with advanced pancreatic cancer did not detect survival benefit with the addition of HCQ to gemcitabine and nab‐paclitaxel,^19^, ^32^ however, a dedicated subgroup analysis to SMAD4 has not been performed. Additional studies according to SMAD4 mutational status could be considered to explore possible survival benefits in patients with SMAD4 loss.

The biologic effects of SMAD4 in cancer are mediated through TGF‐β signaling.^38^ TGF‐β has antiproliferative effects at early stages of cancer, but promotes carcinogenesis and epithelial to mesenchymal transition at later stages.^39^, ^40^ Similarly, autophagy is a double‐edged sword, serving a tumor suppressive function to regulate intracellular damage and apoptosis in normal or premalignant cells or early cancers.^41^ However, in established tumors with hypoxia and nutrient deprivation, autophagy promotes cell survival and tumor growth.^4^ Given the critical association between SMAD4 and TGF‐β, it would be interesting to associate HCQ response with TGF‐β levels. Unfortunately, TGF‐β was not measured in the current retrospective analysis, but warrants further prospective study.

This study is limited by its retrospective nature. The use of combined data from two different chemotherapy regimens and durations from the included prospective clinical trials also confounds our findings. As a result, these data must be interpreted with caution and conclusions are limited. Prospective studies on the role of autophagy inhibition and SMAD4 loss in pancreatic cancer are warranted.

## CONCLUSIONS

Prognosis in pancreatic adenocarcinoma (PDA) is worsened by loss of the tumor suppressor gene SMAD4. SMAD4‐deficient PDA escape radiotherapy and chemotherapy by upregulation of autophagy. In patients with SMAD4 loss, the addition of HCQ to neoadjuvant chemotherapy improved R0 resection rates and resulted in higher degree of histopathologic response. Patients with SMAD4 who received HCQ with neoadjuvant chemotherapy also displayed improved DFS and OS trends, although significance was not met. Further study of autophagy inhibition with HCQ in PDA with SMAD4 loss is warranted.

## CONFLICT OF INTEREST

All other authors declared no competing interests for this work.

## AUTHOR CONTRIBUTIONS

N.F., S.W., and B.B. wrote the manuscript. N.F. and B.B. designed the research. R.R., M.H., A.Z., M.L., N.B., A.S., H.Z., and B.B. performed the research. S.W. analyzed the data. M.H., A.Z., M.L., N.B., A.S., H.Z., and B.B. contributed new reagents/analytical tools.

## ETHICS APPROVAL AND CONSENT TO PARTICIPATE

Institutional review board approval was obtained from the University of Pittsburgh for the clinical trials analyzed in the current work (PRO10010028 and PRO13080444). The trials were registered with the National Cancer Institute (NCT01128296 and NCT01978184). All patients signed informed consent prior to participation. Both trial protocols and consent forms included approval for analysis of tissue specimens and correlation with oncologic outcomes as performed in the current study.

## CONSENT FOR PUBLICATION

The authors consent to publication of this material by the Journal of Clinical and Translational Science. An abstract including these findings has previously been published in the Journal of Clinical Oncology (https://doi.org/10.1200/JCO.2020.38.4_suppl.761 Journal of Clinical Oncology 38, no. 4_suppl [February 1, 2020] 761). The authors guarantee that this manuscript has not been previously published elsewhere.

## Supporting information

Fig S1

Fig S2

Fig S3

Fig S4

Table S1

Table S2

Table S3

## Data Availability

The data that support the findings of this study are available from the corresponding author, B.B., upon reasonable request.

## Footnotes

1 Bold indicates the values *p* < 0.05.
