# Supplementary figures and images for: SMAD4 loss is associated with response to neoadjuvant chemotherapy plus hydroxychloroquine in patients with pancreatic adenocarcinoma
Source: Clin Transl Sci. 2021 May 18;14(5):1822–9. doi: 10.1111/cts.13029 (PMC8504806; doi:10.1111/cts.13029)

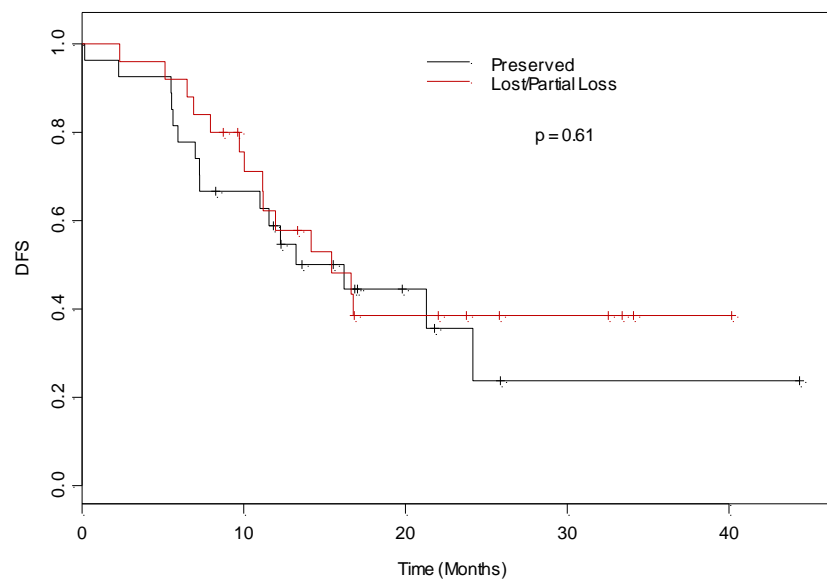

**Supplemental Figure 1:** Disease free survival stratified by SMAD4 in HCQ treated patients (n=52).

Supplement: Supplementary file 1 — Fig S1 [file CTS-14-1822-s006.pdf]

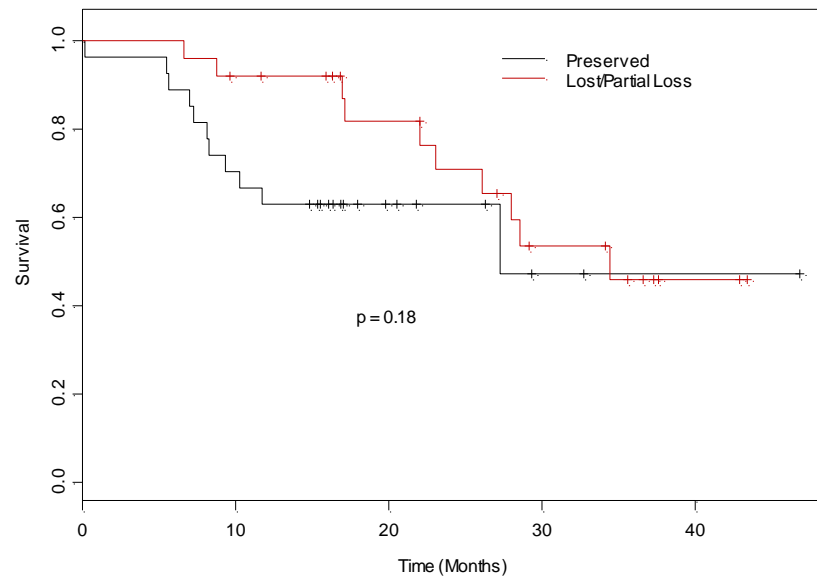

**Supplemental Figure 2:** Overall survival stratified by SMAD4 in HCQ treated patients (n=52).

Supplement: Supplementary file 2 — Fig S2 [file CTS-14-1822-s004.pdf]

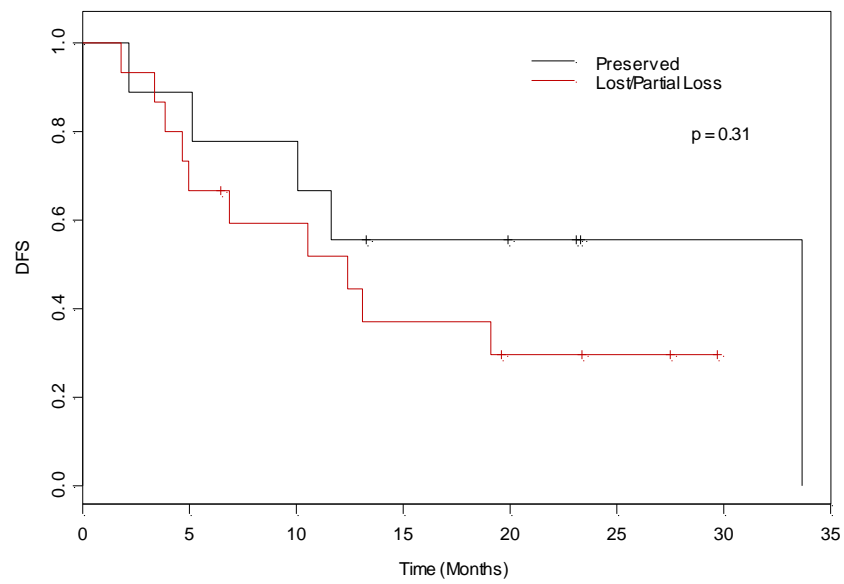

**Supplemental Figure 3:** Disease free survival stratified by SMAD4 in Control patients (n=24).

Supplement: Supplementary file 3 — Fig S3 [file CTS-14-1822-s007.pdf]

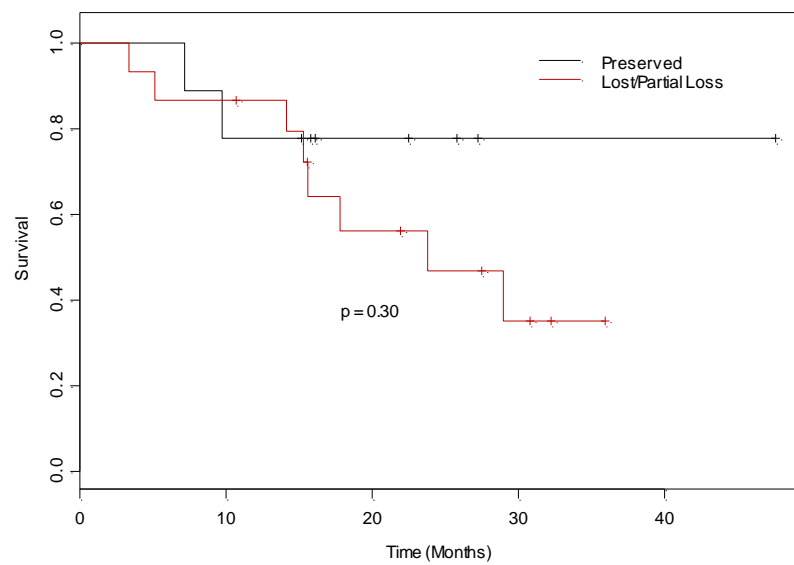

**Supplemental Figure 4:** Overall survival stratified by SMAD4 in Control patients (n=24).

Supplement: Supplementary file 4 — Fig S4 [file CTS-14-1822-s003.pdf]
