# Supplementary material for: SMAD4 loss is associated with response to neoadjuvant chemotherapy plus hydroxychloroquine in patients with pancreatic adenocarcinoma
Source: Clin Transl Sci. 2021 May 18;14(5):1822–9. doi: 10.1111/cts.13029 (PMC8504806; doi:10.1111/cts.13029)
Supplement: Supplementary file 5 — Table S1 [file CTS-14-1822-s001.docx]

**Supplemental Table 1. Outcomes in Control (Chemotherapy alone) group according to SMAD4 status:**

| **Control Group (n=24)** | **SMAD4 Status** | |  |
| --- | --- | --- | --- |
|  | **Preserved** | **Partial Loss /Lost** |  |
| **Total n, (%)** | 9 (38) | 15 (62) |  |
| **Decrease in Ca 19-9 (%)** |  |  |  |
| <50% | 2 (40) | 6 (54.5) | 0.85 |
| ≥50-74% | 2 (40) | 2 (18.2) |  |
| ≥75-89% | 1 (20) | 2 (18.2) |  |
| ≥90% | 0 | 1 (9.1) |  |
| **Evans Grade Histopathologic Response (%)** |  |  |  |
| 1 | 2 (22) | 5 (33) | 0.99 |
| ≥2A | 7 (78) | 10 (67) |  |
| **R0 Resection (%)** | 8 (89) | 9 (60) | 0.19 |
| **Median DFS,** months (95%CI)* | 33.7 (10.1-40+) | 12.4 (5.0-40+) | 0.31 |
| **Median OS**, months (95%CI)* | 40+ (39 - 40+) | 23.8 (15.6 -40+) | 0.30 |
| *from KM-method and log-rank test |  |  |  |
