# Supplementary material for: SMAD4 loss is associated with response to neoadjuvant chemotherapy plus hydroxychloroquine in patients with pancreatic adenocarcinoma
Source: Clin Transl Sci. 2021 May 18;14(5):1822–9. doi: 10.1111/cts.13029 (PMC8504806; doi:10.1111/cts.13029)
Supplement: Supplementary file 6 — Table S2 [file CTS-14-1822-s005.docx]

***Supplemental Table 2. Outcomes in SMAD4 loss patients according to HCQ Treatment***

|  |  | **Chemotherapy Alone (n=15)** | **HCQ + Chemotherapy**  **(n=25)** | **p-value** |
| --- | --- | --- | --- | --- |
| **Evans Grade**  **Histopathologic**  **Response (%)** | 1 | 5 (33.3%) | 6 (24%) | 0.72 |
|  | ≥2A | 10 (66.7%) | 19 (76%) |  |
| **R0 Resection (%)** | No | 6 (40%) | 2 (8%) | **0.036** |
|  | Yes | 9 (60%) | 23 (92%) |  |
| **Median OS (mon)** | | 23.8 (15.6 -40+) | 34.4 (26.1 - 40+) | 0.21 |
| **Median DFS (mon)** | | 12.4 (5.0-40+) | 15.4 (11.2 – 40+) | 0.34 |
