# Supplementary material for: SMAD4 loss is associated with response to neoadjuvant chemotherapy plus hydroxychloroquine in patients with pancreatic adenocarcinoma
Source: Clin Transl Sci. 2021 May 18;14(5):1822–9. doi: 10.1111/cts.13029 (PMC8504806; doi:10.1111/cts.13029)
Supplement: Supplementary file 7 — Table S3 [file CTS-14-1822-s002.docx]

***Supplemental Table 3. Outcomes in SMAD4 preserved patients according to HCQ Treatment***

|  |  | **Chemotherapy Alone (n=9)** | **HCQ + Chemotherapy**  **(n=27)** | **p-value** |
| --- | --- | --- | --- | --- |
| **Evans Grade**  **Histopathologic**  **Response (%)** | 1 | 2 (22.2%) | 17 (63%) | **0.055** |
|  | ≥2A | 7 (77.8%) | 10 (37%) |  |
| **R0 Resection (%)** | No | 1 (11.1%) | 9 (33.3%) | **0.39** |
|  | Yes | 8 (88.9%) | 18 (66.7%) |  |
| **Median OS (mon)** | | 40+ (39 - 40+) | 27.3 (11.7- 40+) | 0.36 |
| **Median DFS (mon)** | | 33.7 (10.1-40+) | 16.2 (11.0 – 40+) | 0.70 |
